# The effect of teacher self-efficacy, teacher resilience, and emotion regulation on teacher burnout: a mediation model

**DOI:** 10.3389/fpsyg.2023.1185079

**Published:** 2023-08-24

**Authors:** Shanshan Li

**Affiliations:** ^1^Faculty of Education, Qufu Normal University, Qufu, China; ^2^Shandong Sport University, Jinan, China

**Keywords:** self-efficacy, teacher resilience, emotion regulation, EFL, burnout

## Abstract

**Introduction:**

This research aimed to explore the relationships among teacher self-efficacy, teacher resilience, emotion regulation, and teacher burnout within the context of Chinese English as a foreign language (EFL) teachers.

**Methods:**

A sample of 638 Chinese EFL teachers participated in this study. They completed self-report assessments for teacher self-efficacy, teacher resilience, emotion regulation, and teacher burnout. Confirmatory factor analysis was conducted to establish the construct validity of the measurement tools. Subsequently, structural equation modeling was utilized to assess the proposed structural model.

**Results:**

The results of the study revealed significant insights. Teacher self-efficacy and resilience exhibited direct and negative associations with teacher burnout. Additionally, an interesting finding emerged where teacher emotion regulation indirectly affected teacher burnout, mediated by teacher resilience. The analysis supported the suitability of the partial mediation model as the best-fit representation of the relationships.

**Discussion:**

The findings of this study provide valuable implications for EFL teaching programs. The negative connections between teacher self-efficacy, resilience, and burnout highlight the importance of nurturing these factors to mitigate burnout risk. The discovered mediation effect of teacher resilience emphasizes the role of emotion regulation in promoting teachers’ overall well-being. These outcomes collectively contribute to the understanding of teacher dynamics and suggest potential avenues for targeted interventions.

## Introduction

Instructors are regarded as key players among the variety of educational system stakeholders because they have the power to influence both individual students’ achievement and the system’s overall performance (Darling-Hammond et al., 2020). As one of the important teacher variables, burnout is viewed as the inability to manage work-related anxiety, deteriorating social relationships, sustained exhaustion, and decreased interest in the profession (Li et al., 2021). Teacher burnout is a pervasive problem that has been widely studied in educational research (Chang, 2009). Burnout among teachers is a significant concern due to its negative impact on teacher well-being, job satisfaction, and student outcomes (Hakanen et al., 2006; Chang, 2013). In recent years, the significance of teacher resilience and self-efficacy has been recognized as essential components of teacher well-being and the prevention of teacher burnout (Daniilidou et al., 2020). In teacher training programs, teachers’ burnout and stress are crucial because it is believed that they may be important contributing factors to teachers’ attrition (Gallant and Riley, 2017; Amitai and Van Houtte, 2022).

Self-efficacy, as another variable, is based on the social cognitive theory, which highlights the development and use of human agency and the idea that individuals can have some control over their behavior (Bandura, 2006). According to the social cognitive theory, self-efficacy of teachers can be characterized as a person’s confidence in their own ability to organize, plan, and perform activities necessary to achieve specific educational objectives. Teacher self-efficacy pertains to a teacher’s belief in their own ability to successfully carry out specific teaching tasks and responsibilities (Tschannen-Moran et al., 1998). It is an important factor in determining a teacher’s performance, job satisfaction, and overall well-being (Tschannen-Moran and Hoy, 2001). Studies have shown that teachers with high levels of self-efficacy are more effective in their instructional practices and are more likely to persist in the face of challenges (Skaalvik and Skaalvik, 2014; Han and Wang, 2021; Liu et al., 2021). They also tend to be more engaged with their students, have better relationships with their colleagues, and experience less stress and burnout (Zee and Koomen, 2016).

Teacher resilience is an important factor that can contribute to reducing teacher burnout (Polat and İskender, 2018). Resilience is the ability to adapt and cope with challenging situations, such as heavy workloads, difficult students, and negative school environments (Mansfield et al., 2016). Resilient teachers are better able to bounce back from setbacks and maintain their motivation and energy levels. Research has shown that resilient teachers are more likely to have a positive attitude toward their work, experience less stress, and have better job satisfaction than less resilient teachers (Daniilidou et al., 2020). Resilient teachers also tend to have better student outcomes, such as improved academic performance and fewer behavior problems. There are several factors that can contribute to teacher resilience, such as social support from colleagues and administrators, positive teacher-student relationships, and effective coping strategies (Beltman et al., 2011; Liu and Chu, 2022). However, it is important to note that resilience is not a fixed trait and can be developed through training and interventions. Given the importance of resilience in preventing teacher burnout, it is crucial to understand the factors that can promote resilience among teachers, especially those working in challenging environments such as Chinese EFL teachers (Chu et al., 2021).

The other variable under investigation in this study is emotion regulation, which is concerned with one’s capacity to manage, alter, and regulate the awareness and conveyance of emotions brought on by both internal and external variables (Wijaya, 2021; Zhao, 2021). It is a method by which individuals try to influence the emotional events they have in order to further their own goals (Colombo et al., 2021). In the teaching profession, emotions, intra-psychological factors, and elements all play a significant role. As a result, teachers’ performance and academic achievement are heavily reliant on their capacity to recognize and manage these feelings (Zhao, 2021). Therefore, it can be concluded that instructors’ ability to control and maintain emotional experiences in the classroom is referred to as teacher emotion regulation (Wang and Ye, 2021). This management may consist of how the teachers perceive, express, alter, maintain, and create emotional interactions.

Although there is a great deal of literature on the psychological aspects of teachers, more empirical research is required to fully understand the elements that affect teachers’ professional careers. Despite the importance of these constructs, research examining the associations among teacher self-efficacy, resilience, emotion regulation, and burnout in the context of Chinese EFL teachers is limited. Therefore, the purpose of this study is to investigate the relationships among teacher self-efficacy, resilience, emotion regulation, and burnout among Chinese EFL teachers. Specifically, we aim to determine whether self-efficacy and resilience predict burnout directly and whether emotion regulation has an indirect effect on burnout through the mediation of teacher resilience. Understanding these relationships has important implications for developing interventions to avoid teacher burnout and foster teacher well-being in the context of EFL teaching programs.

## Literature review

### Teacher burnout

Freudenberger (1974) first introduced the idea of “burnout” as a concept relating to the workplace as a kind of reaction to constant workplace stressors, manifesting as a psychological condition defined by one’s decreased emotional state. According to Maslach and Jackson (1981), burnout can be broken down into three subsections: emotional exhaustion, personal accomplishment, and depersonalization. Emotional exhaustion, personal accomplishment, and depersonalization are all essential aspects of burnout. Personal accomplishment relates to a person’s sense of ineffectiveness and inability to accomplish an assignment. Depersonalization is the process by which an individual begins to feel pessimistic about his/her career. Long-term job stress is thought to be the cause of burnout, especially in human service employees like instructors (Jennett et al., 2003). All instructors may experience stress at work, despite the varied causes, and the majority of them manage such tension well. Burnout, however, might be the result of unsuccessful efforts to deal with continuous tension (Jennett et al., 2003). A syndrome of emotional exhaustion, depersonalization, and decreased personal achievement is known as burnout (Maslach et al., 1996). According to Maslach et al. (1996), burnout is primarily defined by emotional exhaustion, while Pines and Aronson (1988) also included physical exhaustion, which is marked by low energy and persistent fatigue. In this sense, emotional exhaustion was defined as a person’s experience of emotional desolation brought on by stresses, tensions, pressures, and work overload related to their employment. In such circumstances, people might constantly feel exhausted and lack the vigor and enthusiasm to meet the challenges of their everyday jobs (Maslach and Leiter, 2016). Depersonalization in the context of teacher burnout refers to negative, pessimistic mindsets and emotions about one’s students or coworkers. Overall, depersonalized people frequently have negative opinions of their jobs and the coworkers they interact with. Reduced personal accomplishment describes a propensity for teachers to hold a negative opinion of themselves, as well as a broader perception that they are no longer performing a useful and significant job. According to research (Lee and Ashforth, 1996), the three components of burnout cannot be combined into a singular measurement, also Schaufeli and Salanova (2007) identify emotional exhaustion and depersonalization as the two main components of burnout. Research across cultural boundaries demonstrates that indicators of teacher burnout predict not only teachers’ motivation and work satisfaction, but also their subjective and objective health. For example, Hakanen et al. (2006) demonstrated that among Finnish instructors, both emotional exhaustion and depersonalization were inversely correlated with self-rated health as well as job performance. According to available studies (Schaufeli and Salanova, 2007), burnout and motivation have a negative relationship. In addition, in a study of educators in Hong Kong, Leung and Lee (2006) discovered that the exhaustion component of burnout indicated teachers’ intentions to leave the profession. Several investigations have shown a moderate interplay between burnout and instructor self-efficacy (Friedman and Farber, 1992; Evers et al., 2002). However, Skaalvik and Skaalvik (2007) discovered a significant correlation between instructor self-efficacy and exhaustion using structural equation modelling.

According to research, people working in human service fields like medical, health care, social services, and education frequently experience burnout (Lizano, 2015). Teachers frequently experience exhaustion as a result of the demands of their jobs and other obligations (Hiver and Dörnyei, 2017). Although the relevant literature suggests that problems with the educational environment, such as student misbehavior, work-related stress, a lack of support, interpersonal issues, and role ambiguity, are primarily to blame for the emergence of burnout among teachers (Aloe et al., 2014; Scott, 2019), it is also acknowledged that instructors’ psychological inclinations influence how they approach these unfavorable aspects (Herman et al., 2018). Self-efficacy beliefs are the one psychological element that affects teachers’ abilities to cope with common stressors (Schwarzer and Hallum, 2008). Also, recent investigations have explored the interplay between teachers’ emotions, technostress, and burnout in the context of distance learning during the pandemic (Sulla et al., 2022).

### Teacher self-efficacy

Bandura (1997) defined self-efficacy as the belief that one can successfully accomplish a specific task. The first study on teacher self-efficacy was conducted in the late 1970s by the Rand Corporation, building on the work of Rotter (1966) and, more notably, Bandura’s (1997) social cognitive theory. According to Bandura’s (1997) social cognitive theory, teacher self-efficacy refers to the belief of educators in their own abilities to manage particular teaching tasks at a desired level of quality within a specific context. According to Bandura (2006), individuals are self-organizing, active, self-regulating, and reflective in this definition. According to this viewpoint, self-efficacy influences one’s behaviors and objectives and is impacted by both individual behavior and environmental factors (Schunk and Meece, 2006; Skaalvik and Skaalvik, 2010). Self-efficacy has garnered significant attention in L2 research (Piniel, 2013; Fathi et al., 2023), as efficacy beliefs have been found to exert a profound influence on individuals’ activity choices, level of effort invested, and persistence in the face of challenges. Furthermore, efficacy beliefs shape individuals’ perceptions of opportunities and obstacles they encounter during the language learning process (Bandura, 2006). Efficacy beliefs influence people’s choices of activities, the amount of effort they put into those activities, and how long they will persevere in the face of challenges. Moreover, efficacy beliefs decide how chances and barriers are observed (Bandura, 2006).

According to research on the characteristics of teachers, self-efficacy is favorably associated with work satisfaction (Skaalvik and Skaalvik, 2014), work engagement (Han and Wang, 2021), organizational commitment and negatively correlated with burnout (Waweru et al., 2021). Fathi et al. (2021) reported a negative association between teacher self-efficacy and job burnout. Furthermore, instructors with high self-efficacy observed less inappropriate student behavior and were better able to collaborate with their peers to achieve shared educational goals (Goddard and Kim, 2018). Similar results were found by Skaalvik and Skaalvik (2014) among 2,569 teachers in schools, showing that instructors who feel confident in their ability to do their jobs report higher work satisfaction and less emotional exhaustion. Research demonstrates that instructors with high self-efficacy perceptions foster a high-quality learning environment by designing lessons that challenge students’ abilities, handling students’ misbehaviors skillfully, and making an effort to engage students meaningfully (Tsouloupas et al., 2010).

The importance of teacher self-efficacy in relation to job satisfaction has been supported by recent investigations in the field. For example, Sulla and Rollo (2023) examined the effect of a short course on the rates of praise and on-task behavior among Italian primary school teachers. Their findings implied that a positive relationship between teachers’ self-efficacy and job satisfaction, indicating that higher levels of self-efficacy were associated with increased satisfaction in the teaching profession (Sulla and Rollo, 2023). It is generally accepted that instructors who have higher levels of self-efficacy establish an atmosphere for developing stronger bonds with their students and interacting in ways that support behavioral functioning in students (Hamre et al., 2008). Burić and Macuka (2018) used a sample of Croatian teachers to demonstrate that instructors who were self-efficacious reported greater involvement in their job, more satisfaction, love, and enjoyment, and less exhaustion, despair, and anger toward their students. Choi and Lee (2018) used a mixed-methods approach to investigate the relationship between 190 EFL practitioners’ teaching practices and teacher self-efficacy. The application of teaching practice and general self-efficacy were found to be strongly correlated. Additionally, the findings from the interviews suggested that cultural aspects and some opinions about the best methods to teach English had an impact on the relationship between actual teaching and efficacy beliefs. In a later study, Hoang and Wyatt (2021) emphasized the critical influence of culture and context in forming the self-efficacy beliefs, pedagogical approaches, classroom management, and student behavior management of Vietnamese pre-service teachers.

### Teacher resilience

At first, the term “resilience” was used to describe children’s capacity to overcome hardship and develop as a result of it (Li et al., 2019). Early psychological research on resilience focused primarily on identifying distinct personality types and other protective variables that could lessen the negative effects of demanding life circumstances and promote positive adaptation (Luthar and Cicchetti, 2000; Li and Lv, 2022). According to Richardson (2002), there are three stages to the resilience process: the first involves identifying resilience traits and qualities, such as self-efficacy, that can help instructors overcome challenges; the second involves the adaptation process, in which the person tries to cultivate the resilience traits. According to Richardson (2002), this stage is defined as “disruptive reintegrative process for accessing resilient qualities” (p. 307). The individual successfully completes the third phase, which takes a multidisciplinary approach and is compelled by obstacles to develop strength over issues.

As reported by Bernshausen and Cunningham (2001), the notion of resilience alludes to one’s capacity to recover and move forward after facing challenges. To put it another way, it is the capacity to adjust to challenging circumstances and enhance one’s expertise or skill when dealing with pressures and negative events (Bobek, 2002). As noted by Hong (2012), one of the best methods to reduce the number of EFL teachers leaving their jobs is to increase their resilience using the right techniques. As stated by Mansfield et al. (2016), teacher resilience is an ever-evolving process that involves the interaction of both internal and external resources and allows educators to recover from burdens, harmful stressors, and unpleasant incidents in the classroom. The three aspects of “capacity,” “process,” and “outcome” are included in Beltman’s (2015) multidimensional analysis of teacher resilience. The capacity component focuses on teachers’ ability to use the resources they have available to deal with stressful experiences. Process describes a scenario where teachers’ personal characteristics interact with situational variables to adopt effective tactics in the face of difficulties. The final component, outcome, describes how a resilient teacher performs at the end of their career with a higher level of happiness, satisfaction, dedication, and loyalty.

As an evolving field of positive psychology, people have described teacher resilience differently. According to Bobek (2002), teacher resilience is a dynamic process that reflects a teacher’s ability to adjust to various circumstances and strengthens that ability in the face of unfavorable circumstances. Brunetti (2006) defines resilience as the capacity of educators to persevere with their dedication to teaching and pedagogical techniques even in difficult circumstances and despite repeated obstacles. Based on Oswald et al. (2003), teacher resilience is a result of teacher effectiveness and is defined as the capacity to manage one’s limitations and environmental obstacles, regain strength when facing risks, and maintain well-being. Moreover, Day and Gu (2014) discovered moral courage and ethical principles as resilience-enhancing factors for teachers, and they proposed that the ability to sustain balance and possess a strong sense of dedication, control, and ethical direction within the regular environment where teachers carry out their duties is essential. Finally, Tait (2008) asserted that teacher resilience develops through interaction with the environment in difficult situations, and that it is applicable to both personal capacity and setting.

Howard and Johnson (2004) identified several essential traits that resilient teachers consistently demonstrate, such as a sense of agency, moral purpose, a strong support group, and a sense of accomplishment (p. 12). According to other studies, important characteristics of resilient teachers include having a positive attitude towards their work and high ethical standards (Stanford, 2001), the ability to manage their classroom effectively and develop strong connections with their students (Day, 2008), as well as possessing a good sense of humor (Bobek, 2002). Researchers have studied various facets of teacher resilience in relation to the important contributions of teacher resilience to successful educational achievement (Xie, 2021; Li and Lv, 2022). Also, Li and Lv (2022) aimed to find whether there is a link between EFL teachers’ resilience, emotion regulation, and success in the Chinese setting. The results revealed a direct and favorable relationship between emotion regulation, resilience, and success in EFL teachers. Besides, it was discovered that EFL instructors’ resilience was stronger than their emotion regulation in predicting success. Likewise, Xie (2021) investigated the predictive strength of Chinese EFL instructors’ emotion regulation and resilience in their work engagement. According to the results, both resilience and emotion regulation can significantly predict work engagement. Also, Richards et al. (2016) asserted that teacher resilience is essential in reducing the likelihood of teacher burnout.

### Emotion regulation

Emotion regulation was more emphasized in L2 education with the growing interest in positive psychology and, as a result, greater efforts to uncover factors impacting L2 teachers and learners (Wang et al., 2021). According to Gross (1998), emotions arise as a result of the intensity of a repeated pattern of attention and reaction. When an individual faces a problem, they initially react to it, investigate it, and then elicit a particular emotional response. The impact of emotions can be constructive and advantageous, such as in enhancing decision-making skills. However, emotions can also be detrimental when they lead to “maladaptive cognitive or behavioral biases,” depending on the circumstances. Also, Emotion regulation is mainly motivated by harmful and destructive emotions (Gross, 2015).

This concept has been described in various ways since its emergence. Gross (1998) defines emotion regulation as the way individuals control which emotions they feel, when they feel them, and how they express and experience them. Thompson et al. (2008) suggest that emotion regulation encompasses both internal and external processes that involve evaluating and managing emotions to achieve personal objectives. Cole et al. (1994) describe emotion regulation as the capacity to react to life’s circumstances with a range of emotions in a socially acceptable and adaptable manner, allowing for both spontaneous and delayed reactions as appropriate.

Bielak and Mystkowska-Wiertelak (2022) has categorized emotion regulation into two processes: downregulate and upregulate. The former is used to minimize and regulate the impacts of negative emotions, whereas the latter is used to enhance and amplify good emotions. Given the prevalence of teacher-student interactions in the teaching profession, instructors commonly employ emotion regulation strategies. Li and Lv (2022) suggest that instructors could utilize downregulation methods to mitigate negative emotions, like stress, which could impede students’ motivation, engagement, and achievement. Conversely, Gong et al. (2013) propose the use of emotional upregulation techniques to enhance teaching effectiveness and promote academic accomplishment. Similarly, Sutton (2004) recommends that teachers could employ emotional regulation strategies to foster a supportive teacher-student relationship while modeling an idealized image of an emotionally balanced educator.

Gross (2015) makes distinction between two kinds of emotion regulation: “intrinsic emotion regulation” and “extrinsic emotion regulation.” Intrinsic emotion regulation occurs when an individual, especially an adult, seeks to regulate his/her own emotions. A person attempts to control the feelings of another individual through extrinsic regulation, which has been studied in parent–child interactions. Barthel et al. (2018) have stated that regulation of emotions through external support is more effective than self-regulation, as the human brain expends less energy. In language classrooms, where emotional and vulnerable situations arise during learning, instructors can play a crucial role in helping learners manage their emotions through external support (Gkonou and Miller, 2019; Li and Lv, 2022).

Despite a growing interest in investigating emotion regulation across sciences, few studies have focused on language education, especially language teachers’ emotion regulation (Greenier et al., 2021). In this regard, Benesch (2017) argued that in EFL classes, both instructors’ and pupils’ positive and negative emotions influence learning outcomes, with the former boosting and the latter decreasing learning. According to Golombek and Doran (2014), language instruction is an emotionally demanding occupation because of the significance and weight of different interpersonal relationships in EFL contexts. Various studies in various cultural settings have been performed in light of the interest in EFL teachers’ emotion regulation. According to Gong et al. (2013), Chinese teachers employ various emotion regulation strategies and goals to manage their emotions before and after teaching. Their main objective is to minimize the adverse effects of emotions on students’ learning. In a separate study, Yin (2016) investigated the emotion regulation processes used by Chinese teachers and how they influence their professional goals. The findings indicated that the use of emotion regulation techniques is beneficial for teachers in achieving their career objectives, and this, in turn, can impact their overall well-being.

### The present research

Research has shown that higher levels of self-efficacy are associated with lower levels of burnout in teachers (Bandura, 1977; Caprara et al., 2006). Therefore, teacher self-efficacy was considered as a predictor of teacher burnout in the hypothesized model. Also, teacher resilience refers to a teacher’s ability to adapt to and cope with the demands and challenges of their job, including stressful situations. Research has also shown that higher levels of resilience are associated with lower levels of burnout in teachers (Gu and Day, 2013; Mansfield et al., 2016). Therefore, teacher resilience was also added as a predictor of teacher burnout in our study. Emotion regulation refers to a teacher’s ability to manage their emotions effectively in response to job-related stressors. Teachers who are better able to regulate their emotions are less likely to experience burnout (Gross and John, 2003; Castillo-Gualda et al., 2019). As such, emotion regulation was also added as a predictor of teacher burnout in this study. Additionally, teacher resilience was chosen as a mediator variable based on previous research suggesting that it plays an important role in the relationship between teacher self-efficacy and teacher burnout (Gratacós et al., 2021). Specifically, higher levels of teacher self-efficacy may lead to greater resilience, which in turn can reduce the negative impact of stress and prevent burnout. Additionally, emotion regulation has been found to be positively associated with resilience (e.g., Tugade and Fredrickson, 2004), implying that emotion regulation might also indirectly affect teacher burnout through resilience.

In light of the aforementioned background, the primary objective of this study was to explore the interconnectedness between teacher self-efficacy, teacher resilience, emotion regulation, and teacher burnout among Chinese EFL teachers. Specifically, the study sought to examine the direct impact of teacher self-efficacy and resilience on burnout while also investigating the indirect influence of emotion regulation on burnout through the mediating role of teacher resilience. The selection of EFL teachers as the sample for this study was driven by several reasons. Firstly, by focusing on the impact of teacher self-efficacy, resilience, and emotion regulation on burnout within the EFL context, the study aimed to gain valuable insights into the unique challenges and dynamics faced by EFL teachers. EFL teaching entails distinct characteristics, such as instructing English in non-English-speaking countries, working with diverse cultural and linguistic backgrounds, and operating within educational systems that prioritize English language proficiency (Gan, 2013). Secondly, EFL teachers encounter specific job-related stressors that may differ from those experienced by teachers in other subjects or educational contexts (Prapaisit de Segovia and Hardison, 2009; Liu et al., 2023). They often grapple with high demands in terms of lesson planning, language instruction, and managing language barriers (Shamim, 2008). Moreover, they navigate complex classroom dynamics due to students’ varying language proficiency levels and cultural differences, which can contribute to heightened stress levels and burnout risks. This study aims to contribute to the existing literature on teacher burnout by exploring factors that are unique to the EFL context and have the potential to influence burnout outcomes. In so doing, interventions and support mechanisms can be tailored to address their distinct needs and promote their overall well-being (Talbot and Mercer, 2018; Xiyun et al., 2022). The findings of this study hold practical implications for EFL teacher training programs, professional development initiatives, and policies aimed at mitigating burnout and fostering teacher resilience within EFL settings.

## Method

### Participants

The participants of this study were 638 Chinese EFL teachers who were chosen based on convenience sampling. The demographics of the participants are presented in Table 1. The participants ranged in age from 21 to 62, with a mean age of 34.5 years (SD = 7.63). The majority of the participants were female (70.5%), and most had a bachelor’s degree (64.7%). The participants had an average of 9.8 years of teaching experience (SD = 6.23). The study was conducted in China, where English is instructed as a foreign language in many schools and universities.

**Table 1:** Demographics of participants.

Demographics	*N*	%
Age		
Mean (SD)	638	34.5 (7.63)
Gender		
Female	450	70.5
Male	188	29.5
Educational background		
Bachelor’s degree	413	64.7
Master’s degree	209	32.8
Doctoral degree	16	2.5
Teaching experience		
Mean (SD)	638	9.8 (6.23)

In China, individuals aspiring to become EFL teachers typically pursue a bachelor’s degree in English education or a related field. This undergraduate program typically spans 4 years and equips students with a solid foundation in language teaching methodologies, linguistics, and English literature. Upon completing their bachelor’s degree, some individuals may choose to further their education at the master’s or doctoral level to deepen their expertise in the field.

In primary and secondary schools, Chinese EFL teachers typically have a set number of weekly teaching hours, which can range from 15 to 25 h depending on the specific curriculum and school schedule. In higher education institutions, the teaching workload may vary, with some teachers having fewer teaching hours and more time allocated to research and other academic responsibilities. Additionally, the average number of students per class can differ based on the educational level. In primary and secondary schools, class sizes tend to range from around 30 to 50 students, while in higher education institutions, classes are generally smaller, with an average of 20–30 students per class.

### Instruments

As the participants were English teachers who were proficient in the English language, there was no need for translation of the items. Therefore, the original English versions of the scales were used in our research.

#### Teacher self-efficacy scale

The teacher self-efficacy scale TSES is a 12-item self-report scale developed by Tschannen-Moran and Hoy (2001) to assess teachers’ beliefs in their ability to achieve desired instructional outcomes. Participants rated their level of agreement on a 7-point Likert scale (1 = strongly disagree, 7 = strongly agree). Sample items include “I can get through to the most difficult students in my class.” The questionnaire’s validity and reliability have been confirmed through studies in the literature (e.g., Tsigilis et al., 2010; Duffin et al., 2012; Zhang et al., 2023). These studies provide supporting evidence for the factor structure and psychometric properties of the scale, validating its effectiveness in assessing teacher self-efficacy.

#### Teacher resilience scale

The teacher resilience scale (TRS) is a 25-item self-report measure designed by Connor and Davidson (2003) to assess the personal qualities that help individuals cope with adversity and stress. Participants rated their level of agreement on a 5-point Likert scale from 0 (“not true at all”) to 4 (“true nearly all the time”). A sample item is “When things look hopeless, I do not give up.” The Connor-Davidson Resilience Scale has been validated and refined in studies such as Campbell-Sills and Stein (2007) who verified the psychometric properties and validity of the 10-item version of the Resilience Scale in assessing resilience.

#### Emotion regulation questionnaire

The emotion regulation questionnaire (ERQ) is a 10-item self-report scale developed by Gross and John (2003) to assess individuals’ ability to regulate their emotions in a socially acceptable manner. The questionnaire has two subscales: cognitive reappraisal and expressive suppression. Participants rated their level of agreement on a 7-point Likert scale (1 = strongly disagree, 7 = strongly agree). The validity and reliability of the scale have been established in previous studies (e.g., Sala et al., 2012; Ioannidis and Siegling, 2015). These studies provide strong empirical support for the criterion and incremental validity of the Emotion Regulation Questionnaire, affirming its effectiveness in accurately gauging emotion regulation.

#### Maslach burnout inventory-educators survey

To evaluate participant burnout, the Maslach burnout scale for educators (MBI-ES), validated by Maslach et al. (1996), was utilized. The scale comprises 22 items and measures three factors: emotional exhaustion (9 items), depersonalization (5 items), and reduced personal accomplishment (8 items). The scale uses a seven-point Likert scale ranging from 0 (never) to 6 (every day) to assess teacher burnout levels. The dimensionality and psychometric properties of the Maslach Burnout Inventory have been extensively examined and confirmed through various studies (Aluja et al., 2005; Kokkinos, 2006). These rigorous investigations provide compelling evidence supporting the validity and reliability of the inventory as a robust tool for assessing burnout among school teachers.

## Procedure

The ethical approval was obtained from the Institutional Review Board prior to commencing the study, ensuring the protection of participants’ rights and welfare. The research protocol, including the data collection procedure and informed consent process, underwent thorough review and received approval. To adhere to the approved ethical guidelines, data collection was carried out using Chinese online platforms.

Before participating in the survey, participants were provided with a comprehensive explanation of the study’s purpose and procedures. They were explicitly informed about the voluntary nature of their participation, the anonymity of their responses, and the confidential handling of their data. Furthermore, participants were assured that their decision to participate or withdraw would have no bearing on their professional standing or relationship with the involved institutions.

The survey questionnaire consisted of two parts. The first part gathered demographic information, including age, gender, teaching experience, and educational background. The second part included four validated self-report measures assessing the constructs of interest. Each measure utilized a Likert scale format, with participants indicating their agreement or disagreement with provided statements based on their personal experiences.

Participants were instructed to complete the survey independently and allocate sufficient time to ensure thoughtful and accurate responses. They were also encouraged to seek clarification or ask questions regarding any aspect of the survey if needed. Data collection for this study spanned a two-month period, from January to February 2022. During this timeframe, the online survey was administered to the participants, allowing for data collection within the designated period. Participants were given a specific timeframe to complete the survey, ensuring data collection occurred within the defined data collection period.

### Data analysis

To investigate the relationships among the factors, the researcher used SPSS 23.0 to conduct descriptive and correlation analyses. The study’s hypothesis was tested using Structural Equation Modeling (SEM) in Amos program (version 22.0). First, the measurement model was fitted to the data, and then the underlying structural model was examined. The study used several fit indices, including χ2/df, GFI, CFI, RMSEA, and SRMR, to evaluate the overall fitness of the hypothesized model. A χ2/df of less than 3 with a value of *p* greater than 0.05 was considered good, and GFI and CFI values of 0.90 or higher were indicative of good fit (Hu and Bentler, 1999). Additionally, RMSEA <0.08 and SRMR <0.10 were considered good fit indices (Vandenberg and Lance, 2000).

## Results

Before conducting the analysis, missing data, normality, and outliers were checked. The missing data analysis revealed that less than 1% of data was missing, which was handled using the expectation–maximization algorithm (EM). The normality assumption was tested using the skewness and kurtosis values, which were within the acceptable range of ±2.0. The univariate and multivariate outliers were identified using Mahalanobis distance, and three cases were found to be multivariate outliers, which were then removed from the analysis.

Table 2 presents the outcomes of the descriptive and correlation analyses among the constructs. As seen in the table, teacher resilience has a significant positive correlation with emotion regulation (*r* = 0.49, *p* < 0.01, df = 633) and teacher self-efficacy (*r* = 0.44, *p* < 0.01, df = 633). This indicates that higher levels of teacher resilience are associated with higher levels of emotion regulation and self-efficacy. Additionally, emotion regulation has a significant positive correlation with teacher self-efficacy (*r* = 0.34, *p* < 0.01, df = 633), indicating that higher levels of emotion regulation are associated with higher levels of self-efficacy. Also, teacher burnout has a significant negative correlation with teacher resilience (*r* = −0.56, *p* < 0.01, df = 633), emotion regulation (*r* = −0.36, *p* < 0.01, df = 633), and teacher self-efficacy (*r* = −0.38, *p* < 0.01, df = 633), revealing that higher levels of teacher burnout are associated with lower levels of teacher resilience, emotion regulation, and self-efficacy.

**Table 2:** Descriptive statistics and correlations.

	*M*	SD	1	2	3	4
1. Teacher SE	4.07	0.56	–			
2. Teacher resilience	4.37	0.51	0.44*	–		
3. Emotion regulation	3.58	0.63	0.34*	0.49*	–	
4. Teacher burnout	2.71	0.51	−0.38*	−0.56*	−0.36*	–

*N* = 638. *p < 0.01.

Then, the measurement model was assessed using CFA, which showed that the four-factor model (teacher self-efficacy, teacher resilience, emotion regulation, and teacher burnout) provided a good fit to the data (χ2/df = 1.87, CFI = 0.93, TLI = 0.92, RMSEA = 0.06, SRMR = 0.04). Table 3 presents the results of confirmatory factor analysis, including factor loadings, standard errors, and fit indices for each scale.

**Table 3:** Results of CFA.

Scale	Items	Factor 1	Factor 2	Factor 3	Factor 4
Teacher self-efficacy	TE1	0.85 (0.05)	0.12 (0.03)	−0.04 (0.02)	−0.02 (0.02)
	TE2	0.86 (0.04)	0.09 (0.03)	−0.06 (0.02)	0.01 (0.02)
	TE3	0.81 (0.04)	0.07 (0.03)	−0.09 (0.02)	−0.03 (0.02)
Teacher resilience	TR1	−0.03 (0.03)	0.89 (0.04)	0.03 (0.02)	0.04 (0.02)
	TR2	−0.01 (0.03)	0.87 (0.04)	−0.01 (0.02)	0.01 (0.02)
	TR3	0.02 (0.03)	0.84 (0.04)	0.02 (0.02)	−0.03 (0.02)
Emotion regulation	ER1	−0.05 (0.03)	−0.06 (0.03)	0.80 (0.04)	0.06 (0.02)
	ER2	−0.01 (0.03)	−0.03 (0.03)	0.86 (0.04)	0.03 (0.02)
	ER3	−0.07 (0.03)	0.02 (0.03)	0.84 (0.04)	−0.01 (0.02)
Teacher burnout	TB1	−0.01 (0.03)	−0.01 (0.03)	−0.01 (0.03)	0.85 (0.04)
	TB2	−0.05 (0.03)	0.01 (0.03)	−0.03 (0.03)	0.86 (0.04)
	TB3	−0.02 (0.03)	−0.02 (0.03)	0.02 (0.03)	0.87 (0.04)

TE, teacher self-efficacy; TR, teacher resilience; ER, emotion regulation; TB, teacher burnout. Standard errors are in parentheses.

Table 4 provides information about the convergent validity and composite reliability of the constructs. An AVE value of 0.6 or higher indicates that at least 60% of the variance in the construct is explained by its indicators, which suggests good convergent validity. Also, a CR value of 0.7 or higher suggests good reliability. According to the table, all the constructs have good convergent validity as their AVE values are above 0.6. Moreover, all the constructs have good internal consistency as their CR values are above 0.85, which suggests that the items within each construct are measuring the same underlying construct in a reliable manner.

**Table 4:** Convergent validity and composite reliability.

Constructs	AVE	CR
Teacher self-efficacy	0.63	0.91
Emotion regulation	0.62	0.89
Teacher resilience	0.55	0.88
Teacher burnout	0.53	0.85

AVE, average variance extracted; CR, composite reliability.

Moreover, as seen in Table 5. Diagonal elements represent the square root of AVE for each construct, which are 0.79, 0.74, 0.79, and 0.73 for teacher self-efficacy, teacher resilience, emotion regulation, and teacher burnout, respectively. Off-diagonal elements represent the correlation coefficients between the constructs. All off-diagonal elements are lower than the diagonal elements, confirming discriminant validity.

**Table 5:** Discriminant validity.

	1	2	3	4
Self-efficacy	0.79			
Emotion regulation	0.44	0.71		
Resilience	0.34	0.49	0.80	
Burnout	−0.38	−0.56	−0.36	0.73

Once the measurement model was validated, several structural models were evaluated to test the hypotheses. The study compared the hypothesized partial mediation model (Model 3) to a full mediation model (Model 2) and a direct model (Model 1), and their respective fit statistics are presented in Table 6. The results revealed that Model 3 had a significantly better fit than both Model 2 (Δdf = 6, Δχ^2^ = 85.57, *p* < 0.001) and Model 1 (Δdf = 5, Δχ^2^ = 259.33, *p* < 0.001), as indicated by the fit indices employed. As a result, Model 3 was deemed to be the most parsimonious fit for the data.

**Table 6:** Comparison of fit indices for three models.

Model	*χ* ^2^	df	Δ*χ*^2^	GFI	CFI	RMSEA	TLI	SRMR
Direct effect (1)	1050.00**	542	–	0.83	0.92	0.05	0.91	0.17
Full mediation (2)	790.67**	537	259.33	0.85	0.96	0.04	0.94	0.06
Partial mediation (3)	705.10**	531	85.57	0.87	0.97	0.03	0.97	0.05

Δχ^2^ indicates the difference in χ^2^ between the current and subsequent model. **p < 0.001.

**Figure 1:**
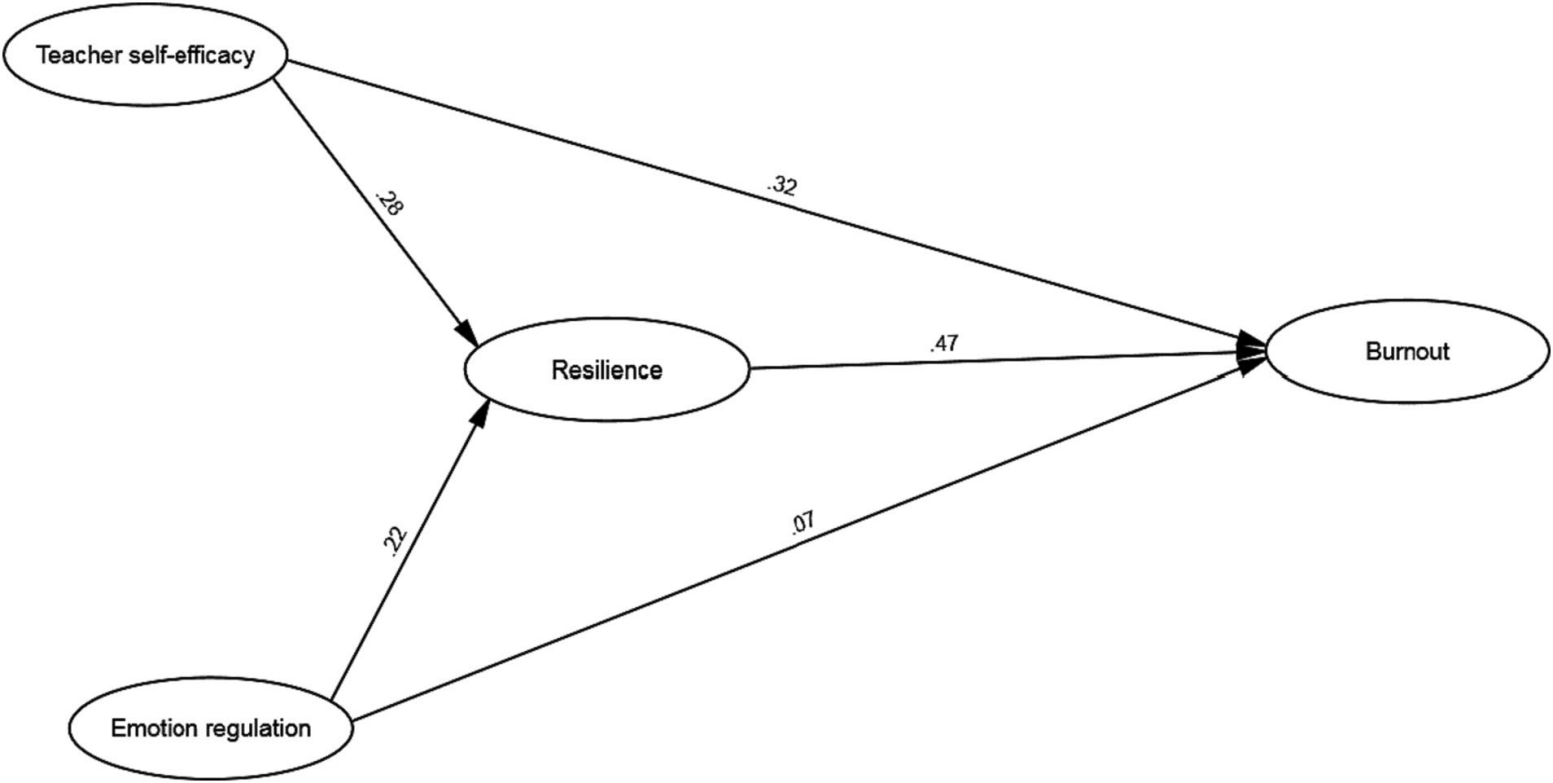
The final partial mediation model.

The final fit model (Partial Mediation) is illustrated in Figure 1, which displays the path and parameter estimates. The path coefficients were significant for all except the path linking teacher emotion regulation and burnout. The structural model reveals that teacher self-efficacy significantly impacted teacher resilience (*β* = 0.32, *p* < 0.01), as did emotion regulation, with a significant positive effect on resilience (*β* = 0.22, *p* < 0.01). Moreover, teacher resilience had a positive association with burnout (*β* = 0.47, *p* < 0.01).

Then, the researcher employed Baron and Kenny's (1986) approach to examine whether teacher resilience acted as a mediator in the relationship between variables. The direct model (Table 7) demonstrated significant path coefficients between self-efficacy, teacher emotion regulation, and burnout (self-efficacy → burnout: 0.40, *p* < 0.001; emotion regulation → burnout: 0.13, *p* < 0.05), thereby fulfilling the first step of Baron and Kenny’s method. The complete mediation model revealed significant path coefficients between self-efficacy and emotion regulation with resilience (self-efficacy → resilience: 0.32, *p* < 0.001; emotion regulation → resilience: 0.25, *p* < 0.01), satisfying the second step of the approach. The partial mediation model demonstrated that teacher resilience partially mediated the link between teacher emotion regulation and burnout. Additionally, teacher emotion regulation had an insignificant path coefficient on burnout, while teacher resilience functioned as a complete mediator between teacher self-efficacy and teacher burnout. Thus, the impact of emotion regulation on teacher resilience influenced burnout.

**Table 7:** Path estimates of structural model.

Standardized path coefficients (*t*-value)			
	Direct effects model	Full mediation model	Partial mediation model
Self-efficacy → burnout	0.40 (5.81***)		0.32 (3.67***)
ER → burnout	0.13 (2.02*)		0.07 (0.78)
Self-efficacy → resilience		0.32 (4.09***)	0.28 (3.98**)
ER → resilience		0.25 (3.08***)	0.22 (2.97**)
Resilience → burnout		0.52 (6.27***)	0.47 (5.22***)

ER, emotion regulation, *p-value < 0.05, **p-value < 0.01, ***p-value < 0.001.

## Discussion

The current study aimed to probe the effect of instructor resilience, instructor self-efficacy, and emotion regulation on teacher burnout among Chinese EFL teachers. The results of testing the hypothesized model indicated some key findings. Firstly, the outcomes of this study demonstrated that teacher self-efficacy directly predicted teacher burnout. This finding supports previous research that has emphasized the importance of teachers’ self-efficacy in increasing teachers’ work engagement and enthusiasm (Skaalvik and Skaalvik, 2007; Federici and Skaalvik, 2011; Perera et al., 2018). The finding is also consistent with previous research that has shown that self-efficacy perceptions are associated with burnout (Friedman, 2003; Schwarzer and Hallum, 2008; Bümen, 2010; Skaalvik and Skaalvik, 2010; Savas et al., 2014; Lauermann and König, 2016; Kim and Burić, 2020; Fathi et al., 2021; Bing et al., 2022). Self-efficacy is concerned with an individual’s belief in their competencies to do specific tasks effectively (Bandura, 1977). From this perspective, teachers with higher levels of self-efficacy might feel more self-assured in their ability to manage challenging situations in the classroom and more competent in their teaching practices. This sense of self-assurance may help teachers to better handle the demands of their job, which may protect them from burnout. Conversely, instructors with low levels of self-efficacy may feel overwhelmed by the demands of their job and may be more likely to experience burnout. As such, EFL instructors’ perceptions about their abilities to use suitable teaching methods, manage their classrooms, and engage students can influence their potential for burnout. In accordance with this finding, Skaalvik and Skaalvik (2007) also concluded that job satisfaction was found to be favorably connected to teacher self-efficacy and negatively correlated to both aspects of teacher burnout, with emotional exhaustion being the most powerful predictor. Another potential explanation for this finding is that whenever EFL Instructors are confident about their abilities and their pedagogical competence to induce instruction, they dedicate more time as well as effort to their profession and are enthusiastically involved in it, thereby experiencing less burnout level.

Furthermore, it was found that resilience could considerably predict EFL instructors’ burnout. This finding is in accordance with previous studies emphasizing the negative association between instructor resilience and burnout (Howard and Johnson, 2004; Beltman et al., 2011; Mansfield et al., 2016; Richards et al., 2016; Polat and İskender, 2018; Daniilidou et al., 2020; Xie, 2021). Resilience is concerned with an individual’s ability to bounce back from adversity and to adapt to changing circumstances (Mansfield et al., 2016). As such, teachers who are more resilient may be better equipped to handle the stressors associated with their job, such as high workloads, difficult students, and challenging classroom environments. This ability to cope with stressors may reduce the risk of burnout by helping instructors to maintain a sense of well-being and job satisfaction. It is argued that more resilient instructors are more inclined to be proficient in managing the environments of the schools and institutions where they work and experience less anxiety as a result. It is also argued that instructors with greater resilience experience less tension and experience higher levels of unity, resulting in a more potent sense of belonging and greater confidence in their abilities to meet standards (Beltman et al., 2011).

Finally, it was revealed that teacher emotion regulation affected teacher burnout directly via the mediation of resilience. This finding is also consistent with previous research that has suggested that emotion regulation is a crucial element in the development of resilience (Bobek, 2002; Tugade and Fredrickson, 2004; Gratacós et al., 2021; Xie, 2021; Li and Lv, 2022). Emotion regulation is concerned with a person’s ability to manage their emotional feedback to different situations (Gross, 1998). In the educational context, teachers who are better able to regulate their emotions may be more resilient in the face of stressors, which might protect them from burnout. Specifically, teachers who are able to regulate their emotions may be better equipped to cope with the demands of their job and may be less probable to experience burnout (Maslach and Leiter, 2016). Additionally, the finding that resilience mediated the relationship between emotion regulation and burnout suggests that interventions aimed at improving emotion regulation skills may be effective in reducing burnout by increasing teachers’ resilience. This finding supports previous studies that have found that emotion regulation is associated with resilience (Kay, 2016; Azpiazu Izaguirre et al., 2021) and that resilience mediates the relationship between emotion regulation and burnout (Brackett et al., 2010; Fried and Chapman, 2012; Chang, 2013; Fiorilli et al., 2017; Castillo-Gualda et al., 2019). These findings suggest that fostering emotion regulation skills may be an effective means of promoting teacher resilience and reducing burnout among EFL teachers.

In accordance with this finding, Bobek (2002) asserted that productive relationships with experienced people help EFL teachers acquire additional insight into approaches to dealing with various difficulties of teaching circumstances, which fosters resilience and emotional competence. One possible justification for this finding is that individuals that take advantage from greater degrees of emotion regulation can experience more work satisfaction, which justifies the positive connection between teacher emotion regulation and success. Teachers who are proficient at assessing, adjusting, and managing their feelings, such as both positive and negative ones, result in fulfilment and pleasure from their work, and they regulate their feelings and tension when facing problems via emotion regulation.

Overall, the findings of this research suggest that teacher self-efficacy, resilience, and emotion regulation are important factors in predicting teacher burnout. The study provides further support for the importance of these factors and highlights the potential benefits of interventions aimed at improving teacher self-efficacy, resilience, and emotion regulation in reducing burnout. This outcome indicates that teacher resilience plays a mediating role in the association between emotion regulation and burnout, but there may be other factors that also contribute to the relationship between these variables. This finding highlights the complexity of the relationship between these variables and suggests that further research is called for to identify other potential mediating factors.

## Conclusion and implications

The purpose of the present study was to broaden the research on psychological factors affecting EFL instructors in Chinese context. For this reason, the effect of teacher resilience, teacher self-efficacy, and emotion regulation on teacher burnout among EFL teachers was investigated. The above-mentioned teacher variables should receive more attention from researchers and educators as these constructs can affect teachers’ exhaustion, resulting in less teacher engagement in classrooms. Generally, this research article highlights the importance of promoting resilience, self-efficacy and emotion regulation among teachers in order to reduce the likelihood of teacher burnout. The results of the study support the theoretical model that teacher self-efficacy and resilience are negatively related to teacher burnout, and teacher emotion regulation has an indirect effect on teacher burnout through the mediation of teacher resilience. This study makes important contributions to the existing literature on the relationship among teacher self-efficacy, resilience, emotion regulation, and burnout. The study confirms the importance of teacher self-efficacy and resilience in protecting against teacher burnout, which is consistent with previous research. Furthermore, the study adds to the literature by showing that teacher emotion regulation also plays a role in preventing burnout. The findings of this study extend the existing theoretical models of teacher burnout and suggest that interventions designed to enhance teacher resilience and emotion regulation may be effective in reducing burnout.

The findings of this study have significant implications for teacher educators and EFL instructors, particularly in the domains of initial and in-service training. These implications underscore the critical importance of prioritizing teachers’ emotional well-being and equipping them with effective strategies for emotion regulation. To this end, teacher training programs should prioritize the inclusion of specific modules that enhance teachers’ understanding of the environmental and psychological factors that influence the effectiveness of emotion regulation techniques. Mentoring programs can play a crucial role by providing opportunities for instructors to learn and apply a diverse range of tactics, gaining valuable insights into the contexts where these strategies yield positive outcomes. Additionally, EFL teacher training should encourage teachers to engage in self-reflection, examining their personal characteristics and preferences that may influence their use of emotion regulation techniques (Farrell, 2016; Xiaojing et al., 2022). By promoting self-reflection, teachers can be empowered to modify and adapt their current practices of emotion regulation, leading to the adoption of more positive and beneficial strategies and enhancing their efficacy in the classroom.

Furthermore, teacher training programs should place a strong emphasis on the development of teachers’ self-efficacy and resilience. These programs should provide teachers with the necessary knowledge and skills to build confidence in effectively managing challenging situations and navigating the demands of their profession. By fostering a sense of resilience, teachers can better cope with stressors and setbacks, reducing the likelihood of burnout and promoting their overall well-being. In addition to teacher training, school administrators and policymakers have a crucial role to play in supporting teachers’ emotional well-being. Educational institutions should ensure that teachers have access to essential resources, such as counseling services and professional development opportunities, to help them effectively manage and regulate their emotions. Furthermore, creating a positive and supportive school climate that encourages open communication and collaboration among teachers can significantly contribute to reducing teacher burnout.

Finally, the current investigation’s findings are constrained by some significant limitations. Firstly, the current investigation was carried out in China, a nation where English is a foreign language. To identify any potential discrepancies in the results, additional EFL/ESL context studies must be conducted in the future. Second, the impacts of contextual factors like age, gender, teaching experience were not studied. To assess the mediating impact that these variables have on the relationship between teacher resilience, teacher self-efficacy, emotion regulation, and teacher burnout, more research on this subject is advised. It is advised that future research use qualitative methods to triangulate results with other quantitative studies in order to obtain a deeper and more accurate assessment of these variables. This will help present a more comprehensive and in-depth view of the relationship between the variables. Additionally, this research made use of information gathered from English instructors at both private institutions and high schools. The impact of these two settings on the self-efficacy, resilience, emotion regulation, and burnout of teachers may be very different. Also, the study was cross-sectional, which limits the ability to draw causal conclusions. Future studies could use longitudinal designs to investigate the causal relationships among teacher self-efficacy, resilience, emotion regulation, and burnout. One limitation of our study is that although the original scales were used in English, it is important to note that locally validated Chinese versions of the scales might have been a more appropriate option for our sample. However, we mitigated this limitation by conducting confirmatory factor analysis on the English scales within our study, ensuring their revalidation and establishing their convergent and discriminant validity.

## Data availability statement

The data analyzed in this study is subject to the following licenses/restrictions: the raw data supporting the conclusions of this article will be made available by the authors, without undue reservation. Requests to access these datasets should be directed to SL, lishanshan@sdpei.edu.cn.

## Ethics statement

The studies involving human participants were reviewed and approved by Faculty of Education, Qufu Normal University. The patients/participants provided their written informed consent to participate in this study.

## Author contributions

The author confirms being the sole contributor of this work and has approved it for publication.

## Conflict of interest

The author declares that the research was conducted in the absence of any commercial or financial relationships that could be construed as a potential conflict of interest.

## Publisher’s note

All claims expressed in this article are solely those of the authors and do not necessarily represent those of their affiliated organizations, or those of the publisher, the editors and the reviewers. Any product that may be evaluated in this article, or claim that may be made by its manufacturer, is not guaranteed or endorsed by the publisher.
